# Electrophoresis-By-Accident in Electrospray Ion Source

**DOI:** 10.1021/acs.analchem.5c08265

**Published:** 2026-04-29

**Authors:** Chikondi Shaba, Pawel L. Urban

**Affiliations:** Department of Chemistry, 34881 National Tsing Hua University, 101, Section 2, Kuang-Fu Rd., Hsinchu 300044, Taiwan

## Abstract

Preparing samples for analysis by mass spectrometry (MS) can be laborious and challenging. Hyphenated separation tools are convenient but costly and require much attention. Here, we present a facile low-cost online MS method for analysis of complex samples. It relies on a standard unmodified electrospray ionization (ESI) source, which incorporates polyether ether ketone tubing connected to a grounded metal union or diverter valve. The sample is infused by a syringe pump, and the flow is stopped. During the stopped-flow stage, ionic species are spontaneously separated due to the electric field between the ESI capillary and the grounded element. The flow is then restarted, and the separated species pass to the ion source. The signals of analytes are enhanced with respect to the signals obtained during direct infusion. This enhancement is attributed to the microseparation of analyte and matrix ions occurring in the sample flow line section. The signal change factors ranged from 0.74 to 295. The reported intrinsic property of ESI—operated in a run-stop-run sequence—has broad applicability in analysis of metabolites, peptides, and proteins in complex matrices. Given the tens of thousands of ESI-MS systems used worldwide, this fundamental finding could have broad applicability.

## Graphical abstract

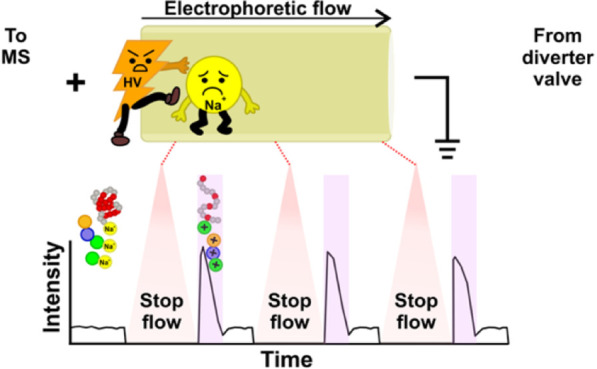

## Introduction

Mass spectrometry (MS)—in many cases—provides the means to perform chemical analyses with high sensitivity.^1^ Electrospray ionization (ESI) enables introduction of liquid-phase analytes with low and high molecular weights to MS.^2^ However, sensitivity of ESI-MS is compromised by undesirable matrix effects.^3,4^ Such matrix effects are often caused by ubiquitous inorganic ions.^5–7^ In order to minimize the matrix effect, samples are treated manually in laborious and time-consuming protocols.^8^ Automation is an option to eliminate human labor^9–11^ but it is costly and not available to every laboratory. In either case, the amounts of pretreated samples need to be relatively large. Complex samples can be analyzed by hyphenated techniques such as liquid chromatography (LC)-ESI-MS^12,13^ or capillary electrophoresis (CE)-ESI-MS.^14^ Both techniques require specialized and costly equipment, much expertise to operate, and careful optimization, while the analyses are time-consuming. One of the special abilities of CE is online preconcentration.^15,16^ Although CE-ESI-MS offers high separation efficiency, challenges related to the ruggedness of CE-MS coupling persist.^17^ Sheath-flow interfaces are generally more reliable but less sensitive than sheathless interfaces.^17–19^ In fact, very few laboratories have specialized in sheathless CE-MS analysis (e.g., Olivares et al.,^20^ Moini,^17^ Hirayama et al.,^21^ and van Mever et al.^22^), and CE-MS has not become the mainstream platform in MS applications related to systems biology. Applying a voltage to a sample solution containing analytes and interferents, such as inorganic salts, establishes an electric field that induces differential migration.^23,24^ The interferents migrate away from the analytes, which have lower electrophoretic mobility,^23^ while also concentrating the analytes at zones of conductivity discontinuity, effectively desalting and stacking.^24^ Electrokinetic-based approaches were used for online desalting of protein samples using a special nanoESI emitter with two electrodes and power supplies,^5^ and desalting and stacking of per/polyfluoroalkyl and opioid samples using paper spray MS.^24^ In another ingenious approach, Taylor-Aris dispersion was employed for protein-salt separation, but required longer analysis times due to moderate flow rates.^4,25^ The approaches summarized above require the use of CE-MS apparatus or other additional elements, which are not present in a standard ESI mass spectrometer.

## Experimental Section

An LCMS-9030 Q-ToF mass spectrometer (Shimadzu, Kyoto, Japan) was used for MS data acquisition in the positive ESI mode. A capillary voltage of 4.0 kV was applied during the entire data acquisition. All data were acquired in MS scan mode. The interface temperature and desolvation line temperature were set to 250 °C, and flow rates of the nebulizing, drying, and heating gases were 3.0, 8.0, and 8.0 L min^–1^, respectively. For caffeine, signal intensities were normalized using an isotope-labeled standard caffeine-(trimethyl-^13^C_3_), both obtained from Sigma-Aldrich (St. Louis, MO, USA). All standards and samples were either prepared or diluted with 10% (v/v) aqueous methanol (LC grade) purchased from Merck (Darmstadt, Germany). LC-MS-grade water was purchased from Fisher Scientific (Waltham, MA, USA). A Legato 130 series syringe pump was used for sample infusion into two PEEK tubings connected in series through a grounded diverter valve on the Q-ToF MS. The specifications of the setup are provided in Figure 1.

**Figure 1:**
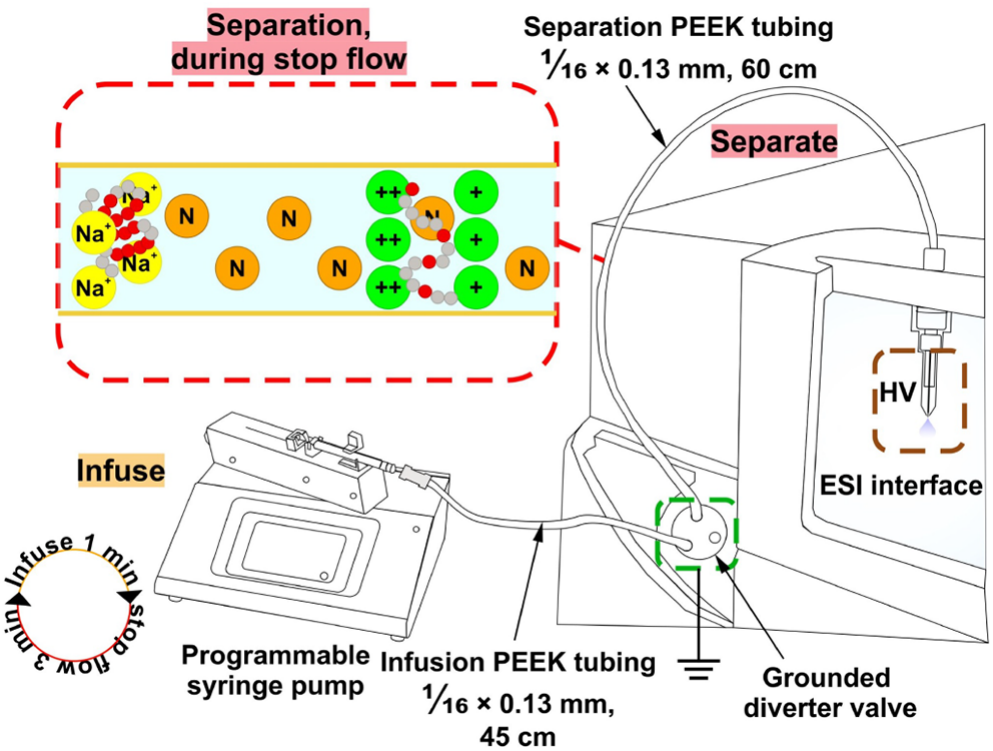
Adaptation of a standard ESI-MS system for electrophoretic desalting and stacking in the sample line.

## Results and Discussion

Here, we demonstrate a simple and rapid approach for online desalting and stacking of samples containing small and large species (bioactive compounds, metabolites, peptides, proteins), which can readily be implemented on a standard ESI mass spectrometer without any physical modification, use of additional power supplies, nESI setup, LC or CE apparatus. The approach takes advantage of the fact that, in many mass spectrometers, the electrospray needle is supplied with a high voltage (several kilovolts) and linked to a grounded metal union or diverter valve via a nonconductive tubing with small inner diameter (cf.^26^ ), which is often made of polyether ether ketone (PEEK; Figure 1). Therefore, an electric field is naturally established in the sample passing through this tubing. That electric field has negligible influence on the MS results during normal infusion of samples to the ESI source, which relies on advective flow. However, in the approach presented here, the sample flow is stopped for a certain period of time (0.5–5.0 min). Modern syringe pumps, used in MS, enable programming such a stopped-flow period in the user interface. During this stopped-flow period, the analyte species and the matrix species—with different electrophoretic mobilities—separate in the electric field developed in the tubing. Following the stopped-flow period, the separated species are eluted through the electrospray needle by turning on the sample pump. As shown later, in some cases, this rudimentary separation is sufficient to minimize interference caused by presence of salt and boost MS signals.

Figure 2 presents typical extracted ion currents (EICs) recorded during elution of a solution containing a cocktail of lower-molecular-weight compounds in the concentration range of 2 × 10^–6^ to 1 × 10^–4^ M and in the presence of 5 mM sodium chloride. A positive correlation is observed between compound concentration and signal intensity. These linear or near-linear responses (Figure S1 and Table S1) demonstrate the approach’s quantitative capability and establish a dynamic range. The presence of sodium chloride provides a controlled saline background, which introduces ionization suppression effects evident in the “before” desalting/stacking region (Figures 2 and S2). The observed trend suggests that the employed desalting and stacking strategy effectively reduces salt-induced interference, allowing for the detection of analytes even at low concentrations. For the small molecules (amino acids), limits of detection (LODs) before ranged from 1.27 × 10^–5^ – 1.78 × 10^–5^ M aside from others being undetectable, and improved to 1.66 × 10^–6^ – 8.58 × 10^–6^ M (Table S1). Furthermore, comparison of signal change factors (SCFs) among compounds (Table S2) reveals analyte-specific variability in susceptibility to ion suppression and desalting. SCFs ranged from 0.74 (lysine) to 295 (acetaminophen), and varied from the absence of detectable signal to measurable signal detection (serine; Table S2). Notably, serine and glutamic acid showed no detectable signals before desalting and stacking but were consistently detected at all concentrations following separation (Figure 2 and Table S2). In addition to sodium chloride, this desalting and stacking strategy was effective for other nonvolatile salts such as lithium chloride and cesium chloride (Figure S2C–E). It was tested across concentrations up to 300 mM for volatile salts (Figure S2A,B) as well as in the presence of various modifiers, i.e., acid, base, and buffer salts (Figure S3). The method remained effective across a neutral-to-acidic pH range in the presence of sodium chloride (Figure S3B). However, ESI emitter clogging was more likely at higher pH. For quantitative analysis, calibration plots should be prepared on the same day because of day-to-day variability of instrument performance (cf. Figure S4). Despite those caveats, the run-stop-run ESI-MS approach is effective at concentrating analytes and minimizing salt interference, particularly for low molecular weight compounds. This is key for applications involving complex matrices, where physiological or environmental salt levels are unavoidable and necessitate sample preparation, for example, in shotgun metabolomics research.

**Figure 2:**
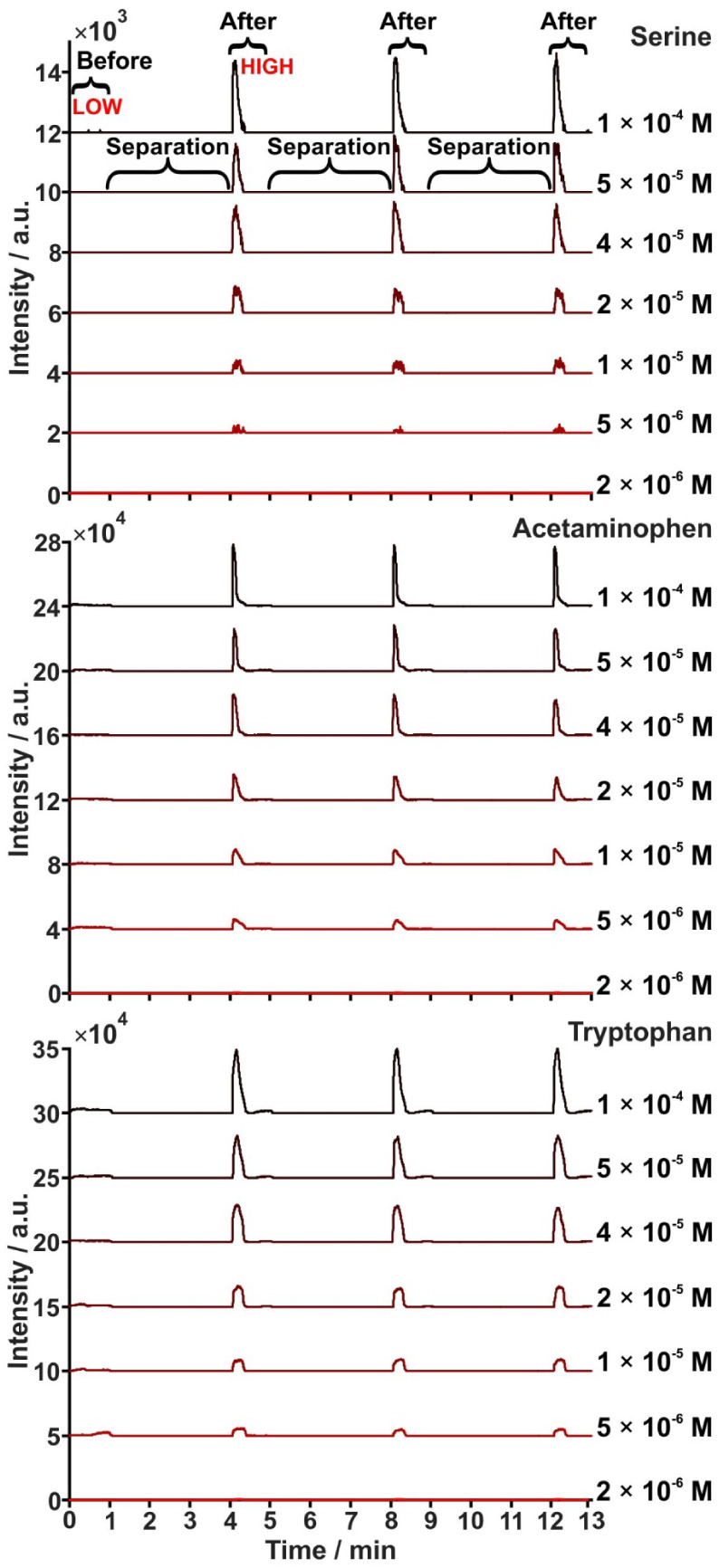
Extracted ion currents of selected compounds in 5 mM sodium chloride at seven increasing concentration levels (2 × 10^‑6^ mol L^‑1^ to 1 × 10^‑4^ mol L^‑1^). Each panel represents a different compound, showing an increasing signal response trend across the concentration range.

Figure 3 illustrates variations in peptide signal intensities under three different salt and acid conditions. In the absence of both sodium chloride and formic acid (modifiers), stacking results in enhancement of signal intensity, particularly pronounced in higher charge states. In the second scenario, when only sodium chloride (no formic acid) is present, signal intensities before desalting are either low or undetectable, probably due to sodium ion-induced ion suppression and adduct formation that diminishes protonated peptide signals. Postseparation (“after”), signals are detectable or increase significantly. In the third condition, when both modifiers are present, there is minimal or no enhancement of signals before or after separation, with overall intensities lower than those observed in the sodium chloride-only condition after separation. This may result from complex interactions between formic acid and salts, such as clustering or an altered ionic environment, which reduce ionization efficiency despite the protonation-promoting effect of acid as a modifier. Interestingly, certain peptide signals (except HPF 10, higher charge state, *z* = 10) that were absent in the sodium chloride-only samples before desalting became detectable under the combined salt and acid condition. This suggests that formic acid might play a role in moderating charge-state distribution or disrupting salt clustering sufficiently to enable effective ionization. The observations align with well-established phenomena affecting ESI-MS performance.^27^

**Figure 3:**
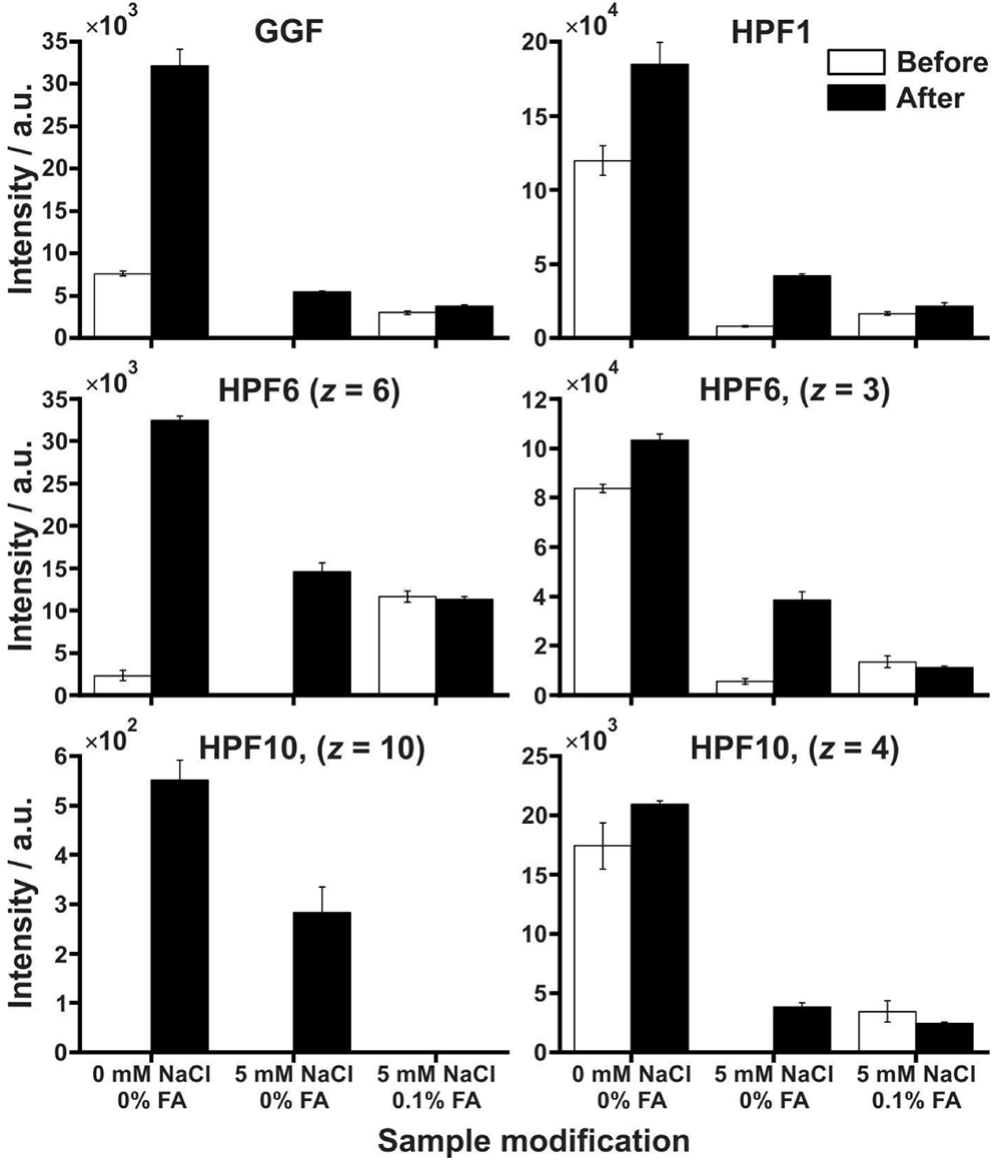
Bar plots of 1 × 10^‑5^ mol L^‑1^ selected peptides in three sample conditions. Each plot shows the signal intensities before (white bar) and after (black bar) desalting and stacking for a peptide and its charge state.

The run-stop-run ESI-MS approach is also suitable for analysis of larger molecular weight species. Figure 4 presents a typical total ion current (TIC) recorded during elution of the 10 μM ubiquitin solution containing 5 mM sodium chloride. The intensity of *m*/*z* 1428.2817 (*z* = 6) rose markedly after the stopped-flow period (Figure 4C), reaching 1738 ± 26 compared to 232 ± 38 (*n* = 3) in the preceding “before” region (Figure 4B). A similar observation was made for cytochrome *c* (Figure S5). Similar to Figure 2, these results reveal the simultaneous desalting and stacking of the proteins in a section of the PEEK tubing adjacent to the electrospray emitter. Before separation, the presence of prominent sodium adduct signals severely complicates spectral interpretation, rendering deconvolution challenging or impossible. This complex adduct pattern contributes to decreased signal clarity. Following the separation period, the “after” spectra exhibit an apparent reduction or elimination of sodium adduct peaks, substantially clarifying the mass spectrum. This improvement enables effective deconvolution, charge-state assignment, and interpretation of high-resolution mass spectrometric data for proteins in saline matrices.

**Figure 4:**
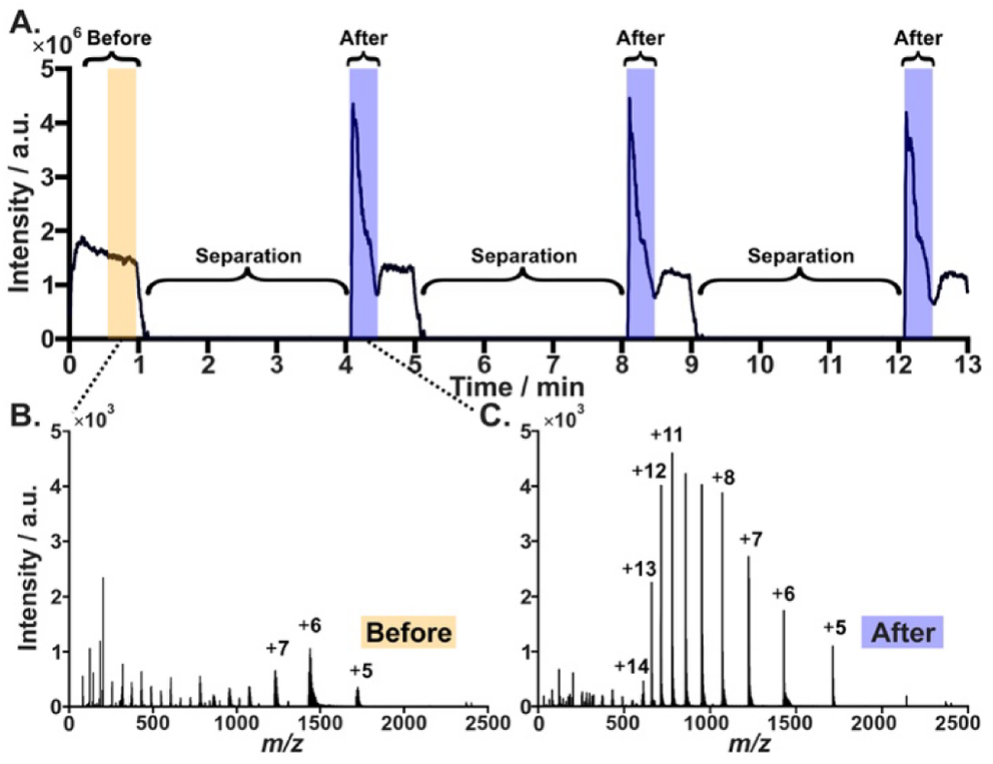
Online desalting and stacking of 1 × 10^‑5^ mol L^‑1^ ubiquitin in 5 mmol L^‑1^ sodium chloride showing signal intensities of the sodiate region (before, orange bar) and the desalted and stacked region (after, blue bar). Total ion current of MS scan range *m*/*z* 20–3000 (A) and representative spectra (B, C) recorded during four independent 1 min syringe infusion runs: the first (before) without incubation, while the subsequent three (after) followed a 3 min incubation (separation).

We have challenged this analytical approach while analyzing real samples (energy drinks). Isotope-labeled caffeine was introduced as an internal standard, it became sodiated in the NaCl solution and, after the incubation period, lost the sodium adduct. This, however, did not affect its use as an internal standard (Figure S6A,B). Linearity and LoD improved with the inclusion of the internal standard (Figure S6A,B and Table S3). A significant improvement (∼6 times) was observed in the solution with lower NaCl concentration, the LoD of caffeine improved from 1.09 × 10^–6^ without normalization to 1.73 × 10^–7^ M with normalization (Table S3). Figure S6C shows that for sugar containing energy drink brand 1 (ED1-S), the less diluted sample yielded a lower caffeine concentration than the more diluted sample. The latter more closely matched the labeled concentration. This is likely due to ion suppression and matrix effects caused by salts and other additives, such as sugar. Thus, in the case of such a complex matrix, a dilute sample is subjected to online desalting, to minimize matrix effect further.

Electrospray emitter functions as an electrolytic cell with electrochemical oxidation occurring at the metal-liquid interface, creating excess positive charge in the solution (low pH).^23,26,28^ The cathodic reactions occur at a grounded electrode, producing OH^–^ (high pH), establishing a pH gradient via electromigration (cf.^23,29^ ). Similarly, an in-syringe electrokinetic method created a pH gradient for isoelectric focusing (IEF) of ampholytes by applying negative voltage to the needle and grounding the plunger.^29^ The current run-stop-run approach, with a positive voltage at the emitter and ground at the diverter valve, may also generate a transient pH gradient through electrochemical reactions at the emitter. However, because of the low stability of the resulting pH gradient (cf.^30^ ), and lack of discrete analyte zones at the start, it is not likely that the IEF mechanism is responsible for the signal enhancement. Nonetheless, the local pH drop can affect the protein folding state and charge state distribution (cf. Figures 4B,C and S5B,C). Furthermore, ionic species are stacked, in a similar way as it happens in CE stacking methods (cf.^31,32^ ), further contributing to the peak intensities. Similar to Wei et al.‘s step-voltage approach,^33^ the run-stop-run ESI-MS approach presented here also halts fluid flow, creating a period for electrophoretic separation. Since fluid flow in normal nESI significantly exceeds electrophoretic mobility,^23,33^ stopping flow allows electrophoretic migration to dominate, enabling separation of low-mobility analytes from high-mobility salt ions^23,33^ before resuming flow for elution. As previously noted, reduced “saturation” in droplets minimizes charge competition and ion suppression, leading to signal enhancement, extended linear dynamic range, and improved sensitivity.^34^ The spatial separation of the interfering salts during the stopped-flow period may minimize electrochemical competition, allowing for efficient production of positively charged analyte species, and improved ionization efficiency.

While the presented approach works as is, on a standard MS instrument, additional optimization experiments (Figure S7A) showed that grounding is essential for signal enhancement, which further corroborates the proposed spontaneous electrophoretic separation mechanism. Grounding loop is present in many commercial systems, and one does not need to introduce modifications in such systems. However, in some systems, a voltage is present at the ion inlet while the ESI capillary is grounded. In those particular cases, an additional modification of the sample flow line is needed following appropriate safety precautions. In our study, with the voltage applied to the ESI capillary, a 60 cm PEEK tubing section provided optimal separation, while a 3 min separation time balanced electrophoretic migration against diffusional broadening (Figure S7).

Comparable to our desalting/stacking approach is the more recently published work on Taylor/non-Taylor dispersion by Maciel et al., which represents a significant advance in accessibility by achieving ultrafast desalting from conventional biological buffers without chromatography or specialized equipment.^35^ Their approach requires two syringes and focuses on a higher mass range while operating at lower sample volume.^35^ In contrast, our approach simplifies the desalting/stacking system by using a single syringe and an ESI conductive capillary (standard setup), and demonstrates applicability across a wide mass range, albeit with a larger sample volume (consistent with standard ESI operation) and certain day-to-day variability.

## Conclusion

Effects of the spontaneous electrophoresis phenomenon discussed here were likely seen by mass spectrometrists previously but possibly regarded as artifacts of sample injection to ESI-MS source. Analysts often encounter unstable mass spectral signals at the beginning of data acquisition.^36^ One typically waits until MS ion current stabilizes before using it for quantitative purposes. Here, we show that the *first peak* recorded after a stopped-flow period can be caused by spontaneous electrophoretic separation in the sample line and can be used for reliable measurements with high sensitivity. The above finding is likely relevant to many chemists using the tens of thousands of ESI-MS systems installed worldwide, and that is why we wish to report it in this eminent outlet.

## Supplementary Material



## Notes

Figures S1–S7: calibration plots of selected compounds; EICs of acetaminophen in increasing concentrations of salts; EICs of acetaminophen in modifiers; day-to-day variability; online desalting and stacking of cytochrome*c*; calibration plots and concentrations of caffeine; optimization; Tables S1–S3: calibration plot parameters and limits of detection; signal change factors of selected compounds; calibration plot parameters and limits of detection of caffeine (PDF)
