# Supplementary material for: Electrophoresis-By-Accident in Electrospray Ion Source
Source: Anal Chem. 2026 Apr 29;98(18):13220–5. doi: 10.1021/acs.analchem.5c08265 (PMC13177287; doi:10.1021/acs.analchem.5c08265)
Supplement: Supplementary file 1 [file ac5c08265_si_001.pdf]

SUPPORTING INFORMATION

Electrophoresis-By-Accident in Electrospray Ion Source

Chikondi Shaba and Pawel L. Urban*

Department of Chemistry, National Tsing Hua University

101, Section 2, Kuang-Fu Rd., Hsinchu, 300044, Taiwan

* Corresponding author:

P.L. Urban (urban@mx.nthu.edu.tw)

Table of Contents:

Figures S1-S7……………………………………………………………………………S-2

Tables S1-S3……………………………………………………………………………….S-9

ADDITIONAL FIGURES

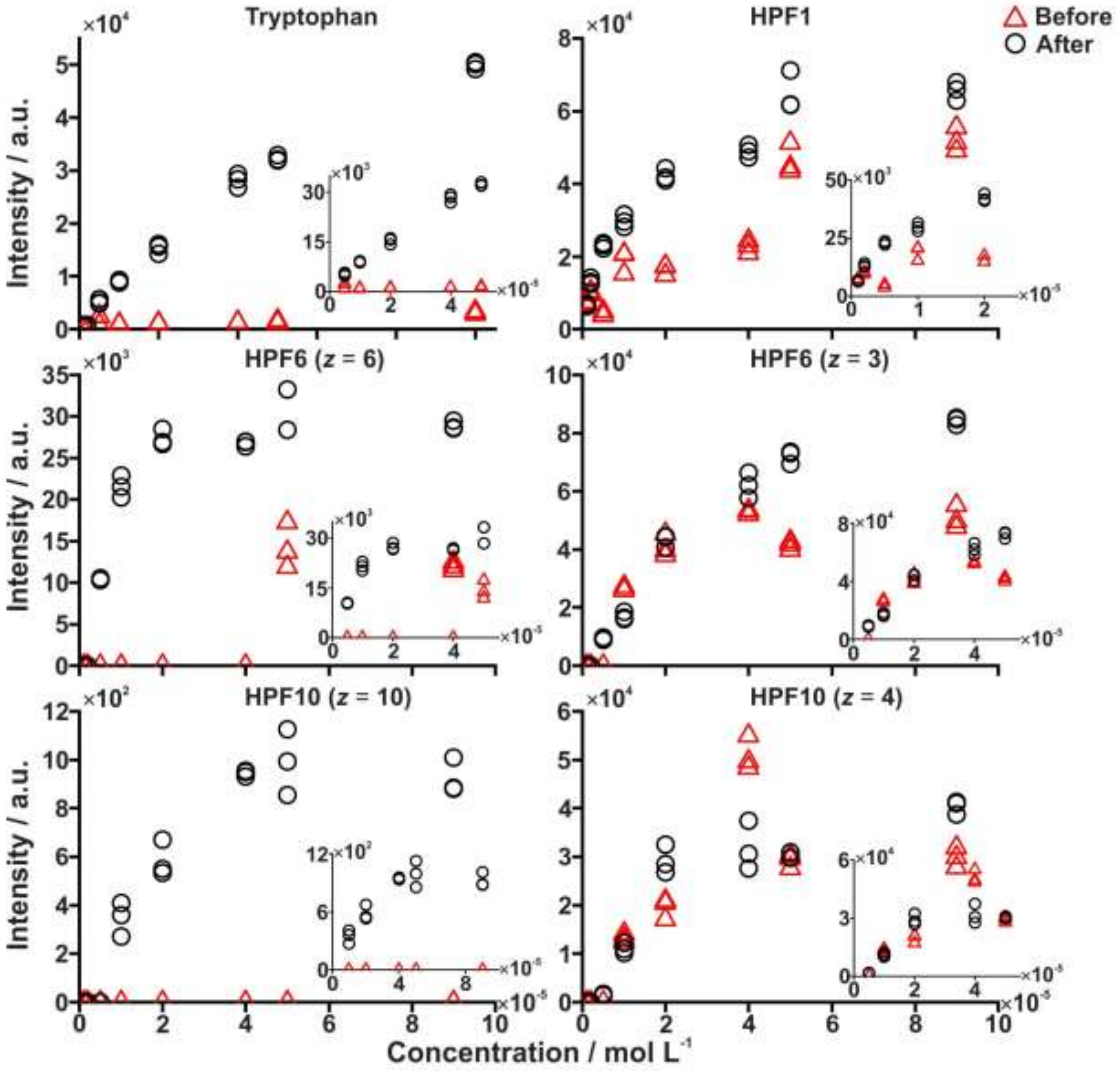

Figure S1. Calibration plots of selected compounds in a solution of 5 mM sodium chloride. Each panel presents a different compound, with an inset showing the linear range of its calibration curve.

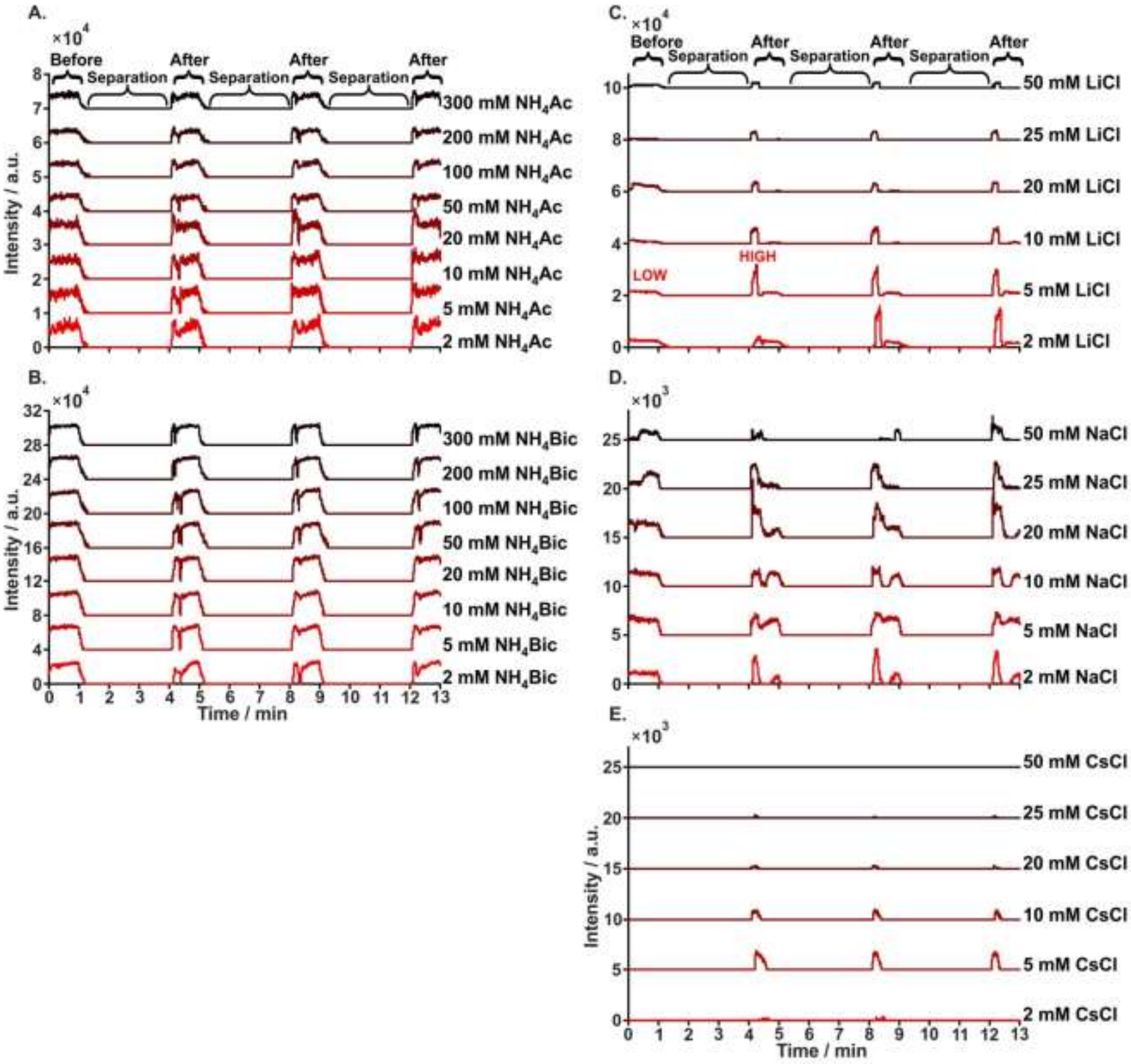

Figure S2. Extracted ion currents of 1×10^{-5} mol L^{-1} tryptophan in the presence of increasing concentrations of volatile (A, B) and non-volatile salts (C-E), prepared separately. Each panel represents a different salt: A, ammonium acetate (NH_4Ac); B, ammonium bicarbonate (NH_4Bic); C, lithium chloride (LiCl); D, sodium chloride (NaCl); and E, cesium chloride (CsCl).

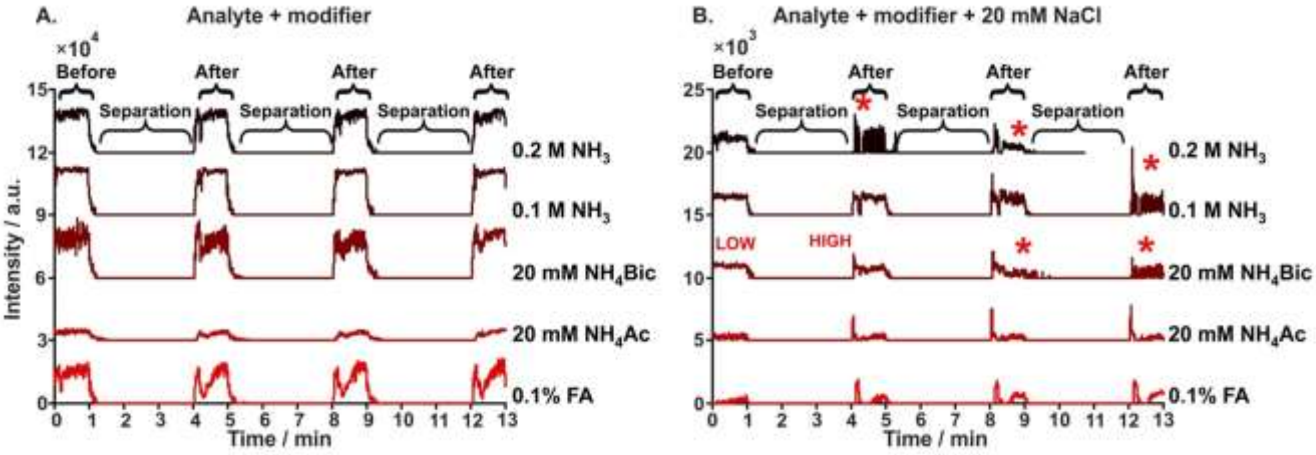

Figure S3. Extracted ion currents of 1×10^{-5} mol L^{-1} tryptophan in the presence of a modifier only (A) and modifier plus 20 mM sodium chloride (B). Each modifier altered the pH of the solution (top to bottom, decreasing pH): 0.2 M ammonia (NH_3), pH ≈ 11.4; 0.1 M ammonia (NH_3), pH ≈ 11.0; 20 mM ammonium bicarbonate (NH_4Bic), pH ≈ 7.6; 20 mM ammonium acetate (NH_4Ac), pH ≈ 6.6; and 0.1% formic acid (FA), pH ≈ 2.4. The symbol * indicates clogging at the emitter during acquisition.

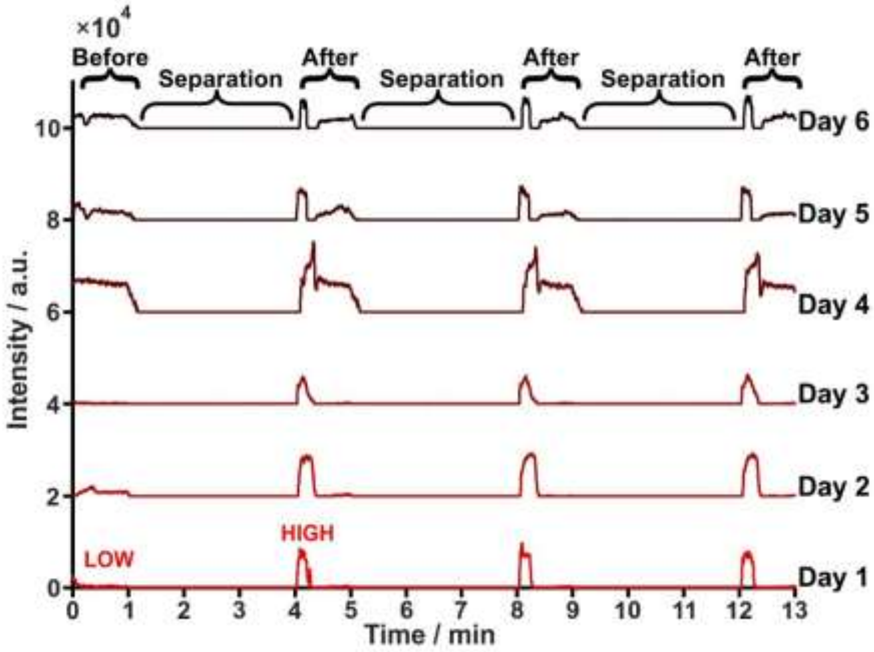

Figure S4. Day-to-day variability represented by extracted ion currents of 1 × 10^{-5} mol L^{-1} tryptophan prepared in a solution of 5 mM sodium chloride. Day 1 was 18 April 2025, day 2 was 22 April 2025, day 3 was 3 June 2025, day 4 was 25 March 2026, day 5 was 11 April 2026, and day 6 was 14 April 2026.

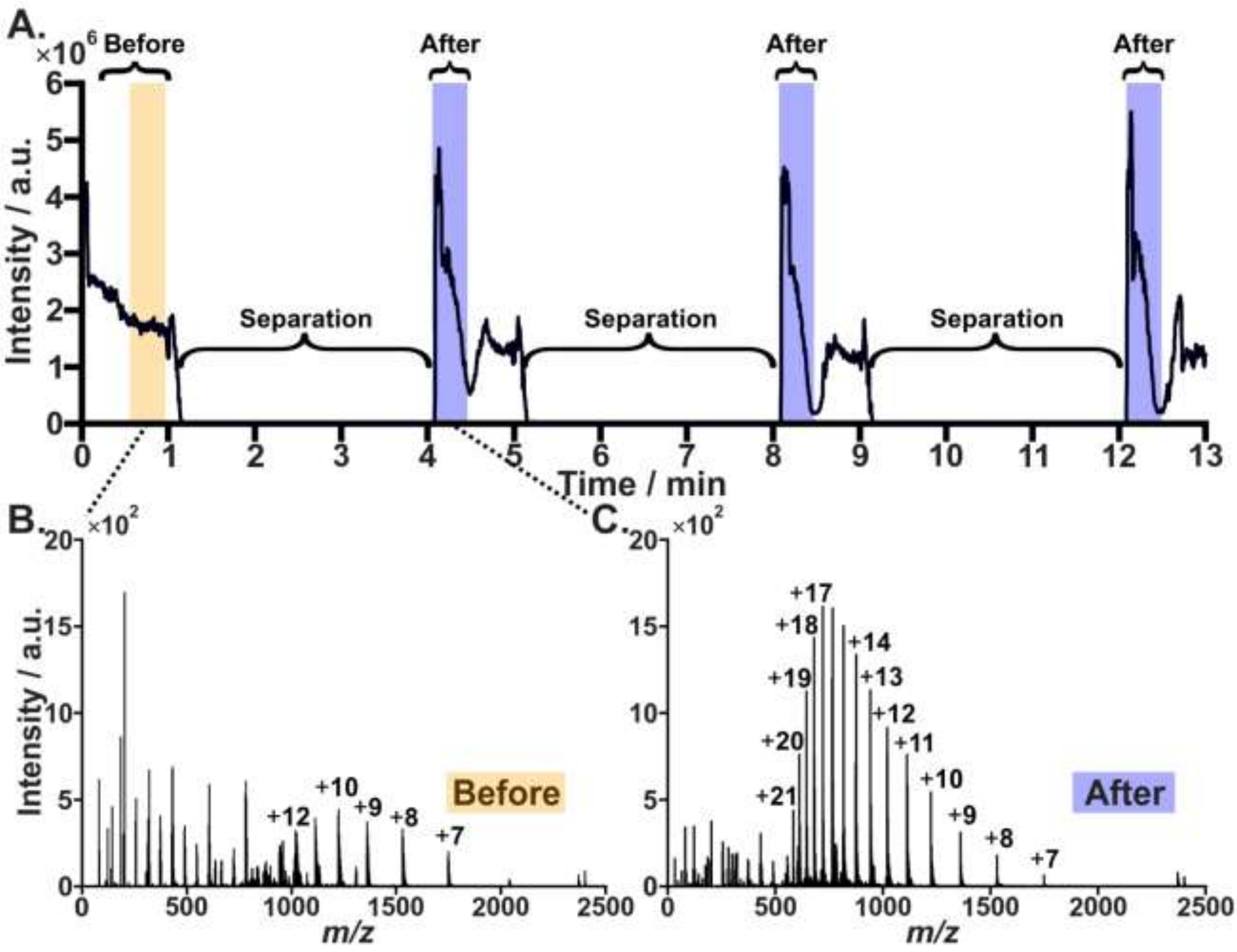

Figure S5. Online desalting and stacking of 1×10^{-5} mol L^{-1} cytochrome *c* in 5 mmol L^{-1} sodium chloride showing signal intensities of the sodiated region (before, orange bar) and the desalted and stacked region (after, blue bar). Total ion current of MS scan range *m/z* 20-3000 (A) and representative spectra (B, C) recorded during four independent 1-min syringe infusion runs: the first (before) without incubation, while the subsequent three (after) followed a 3-min incubation (separation).

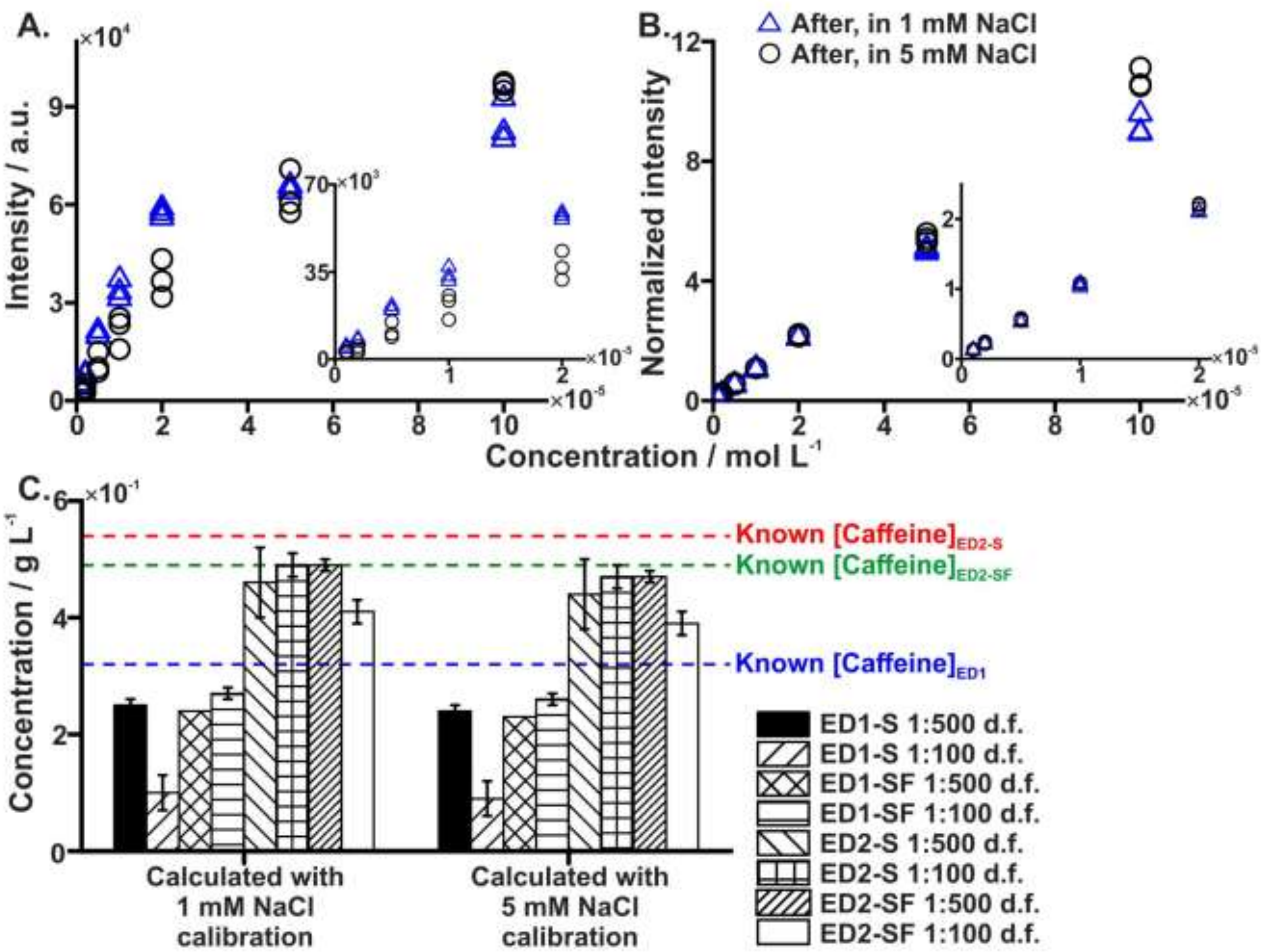

Figure S6. Calibration plots and concentrations of caffeine in solutions of 1 mM and 5 mM sodium chloride. A and B) Each panel presents a different compound with an inset showing the linear range of the corresponding calibration curve. C) Bar plot of experimentally determined concentrations of caffeine in four caffeinated beverages, each diluted 100 and 500 times. The beverages are: energy drink brand 1 (ED1) with sugar (ED1-S) and sugar free (ED1-SF); energy drink brand 2 (ED2) with sugar (ED2-S) and sugar free (ED2-SF); and all contain salt. The reference lines indicate the branded caffeine concentration labeled on the beverage packaging.

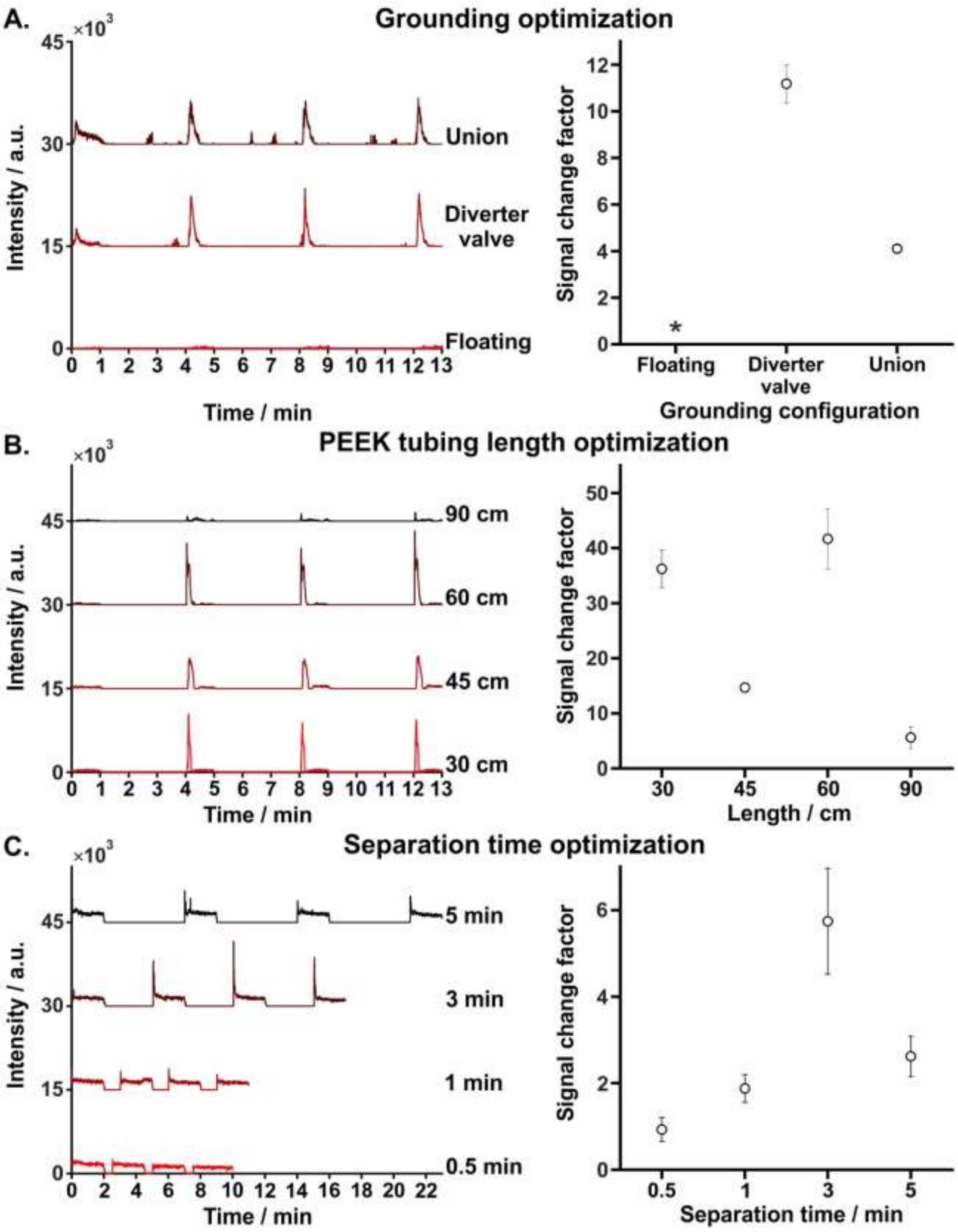

Figure S7. Optimization of grounding configuration (A), PEEK tubing length (B), and separation time (C) using 1×10^{-5} mol L^{-1} acetaminophen in 5 mmol L^{-1} sodium chloride solution. The left column shows extracted ion currents depicting online desalting and stacking, and the right column shows signal change factors (n = 3). The asterisk (∗) indicates the absence of a quantifiable signal before and after separation.

ADDITIONAL TABLES

Table S1. Calibration plot parameters and limits of detection of selected compounds in a solution of 5 mM sodium chloride. The LOD and LOQ values were calculated based on the slope and the intercept error provided by the Origin software.

Compound		Calibration equation (C / mol L^{-1})	R^2	LOD / mol L^{-1}	LOQ / mol L^{-1}
Serine	Before	*Intensity* = (0.00 ± 0.00) C + (0.00 ± 0.00)	*	*	*
	After	*Intensity* = (3.50 ± 0.18) × 10^7 C + (1.07 ± 0.54) × 10^2	0.9682	4.59 × 10^{-6}	9.18 × 10^{-6}
Lysine	Before	*Intensity* = (6.73 ± 0.53) × 10^7 C + (3.06 ± 0.29) × 10^3	0.9259	1.27 × 10^{-5}	2.54 × 10^{-5}
	After	*Intensity* = (7.23 ± 0.38) × 10^7 C + (2.84 ± 0.21) × 10^3	0.9648	8.58 × 10^{-6}	1.72 × 10^{-5}
Glutamic acid	Before	*Intensity* = (0.00 ± 0.00) C + (0.00 ± 0.00)	*	*	*
	After	*Intensity* = (9.46 ± 0.68) × 10^7 C − (7.29 ± 20.64) × 10^1	0.9373	6.55 × 10^{-6}	1.31 × 10^{-5}
Acetaminophen	Before	*Intensity* = (-1.65 ± 0.27) × 10^7 C + (1.06 ± 0.08) × 10^3	0.7480	1.47 × 10^{-5}	2.94 × 10^{-5}
	After	*Intensity* = (5.58 ± 0.24) × 10^8 C + (1.75 ± 0.63) × 10^3	0.9630	3.38 × 10^{-6}	6.76 × 10^{-6}
Histidine	Before	*Intensity* = (1.71 ± 0.33) × 10^8 C + (1.02 ± 0.10) × 10^4	0.6689	1.78 × 10^{-5}	3.56 × 10^{-5}
	After	*Intensity* = (4.21 ± 0.25) × 10^8 C + (8.99 ± 0.75) × 10^3	0.9573	5.34 × 10^{-6}	1.07 × 10^{-5}
Phenyalanine	Before	*Intensity* = (14.47 ± 5.56) × 10^6 C + (1.03 ± 0.17) × 10^3	0.0473	1.14 × 10^{-4}	2.27 × 10^{-4}
	After	*Intensity* = (6.29 ± 0.11) × 10^8 C + (2.65 ± 0.35) × 10^3	0.9957	1.66 × 10^{-6}	3.31 × 10^{-6}
Tryptophan	Before	*Intensity* = (-1.12 ± 7.48) × 10^6 C + (1.40 ± 0.23) × 10^3	0.0017	6.08 × 10^{-4}	1.22 × 10^{-3}
	After	*Intensity* = (6.09 ± 0.15) × 10^8 C + (2.80 ± 0.46) × 10^3	0.9920	2.28 × 10^{-6}	4.55 × 10^{-6}
HPF1	Before	*Intensity* = (5.66 ± 1.65) × 10^8 C + (7.04 ± 1.70) × 10^3	0.4734	9.04 × 10^{-6}	1.18 × 10^{-5}
	After	*Intensity* = (1.74 ± 0.14) × 10^9 C + (9.79 ± 1.44) × 10^3	0.9222	2.49 × 10^{-6}	4.98 × 10^{-6}
HPF6 (z=6)	Before	*Intensity* = (2.38 ± 0.65) × 10^8 C − (3.10 ± 1.99) × 10^3	0.5052	2.50 × 10^{-5}	5.01 × 10^{-5}
	After	*Intensity* = (3.22 ± 0.69) × 10^8 C + (1.52 ± 0.21) × 10^3	0.6228	1.97 × 10^{-5}	3.94 × 10^{-5}
HPF6 (z=5)	Before	*Intensity* = (7.19 ± 1.59) × 10^8 C − (5.80 ± 4.84) × 10^3	0.6111	2.50 × 10^{-5}	2.50 × 10^{-5}
	After	*Intensity* = (9.08 ± 1.05) × 10^8 C + (1.81 ± 0.32) × 10^3	0.8514	1.06 × 10^{-5}	2.11 × 10^{-5}
HPF6 (z=4)	Before	*Intensity* = (7.92 ± 0.78) × 10^8 C − (1.51 ± 2.38) × 10^3	0.8876	9.01 × 10^{-6}	1.80 × 10^{-5}

	After	*Intensity* = (9.44 ± 0.59) × 10^8 *C* + (8.51 ± 1.80) × 10^3	0.9512	5.73 × 10^{-6}	1.15 × 10^{-5}
HPF6 (*z*=3)	Before	*Intensity* = (8.18 ± 1.85) × 10^8 *C* + (1.19 ± 0.56) × 10^4	0.6009	2.06 × 10^{-5}	4.13 × 10^{-5}
	After	*Intensity* = (1.38 ± 0.08) × 10^9 *C* + (6.20 ± 2.58) × 10^3	0.9533	5.60 × 10^{-6}	1.12 × 10^{-5}
HPF10 (*z*=10)	Before	*Intensity* = (0.00 ± 0.00) *C* + (0.00 ± 0.00)	*	*	*
	After	*Intensity* = (6.84 ± 1.78) × 10^6 *C* + (4.71 ± 0.89) × 10^2	0.5322	3.93 × 10^{-5}	7.86 × 10^{-5}
HPF10 (*z*=9)	Before	*Intensity* = (1.01 ± 0.27) × 10^8 *C* − (1.32 ± 0.83) × 10^3	0.5162	2.45 × 10^{-5}	4.90 × 10^{-5}
	After	*Intensity* = (2.96 ± 0.28) × 10^8 *C* + (9.58 ± 0.21) × 10^3	0.8971	8.57 × 10^{-6}	1.71 × 10^{-5}
HPF10 (*z*=8)	Before	*Intensity* = (4.20 ± 0.94) × 10^8 *C* − (5.27 ± 2.85) × 10^3	0.6072	2.04 × 10^{-5}	4.07 × 10^{-5}
	After	*Intensity* = (8.99 ± 0.64) × 10^8 *C* + (2.17 ± 1.93) × 10^3	0.9392	6.44 × 10^{-6}	1.29 × 10^{-5}
HPF10 (*z*=7)	Before	*Intensity* = (7.14 ± 1.34) × 10^8 *C* − (8.10 ± 4.08) × 10^3	0.6858	1.71 × 10^{-5}	3.43 × 10^{-5}
	After	*Intensity* = (1.08 ± 0.08) × 10^9 *C* + (2.23 ± 2.39) × 10^3	0.9361	6.61 × 10^{-6}	1.32 × 10^{-5}
HPF10 (*z*=6)	Before	*Intensity* = (8.22 ± 0.96) × 10^8 *C* − (7.75 ± 2.92) × 10^3	0.8489	1.07 × 10^{-5}	2.14 × 10^{-5}
	After	*Intensity* = (7.87 ± 0.67) × 10^8 *C* + (2.36 ± 2.05) × 10^3	0.9129	7.82 × 10^{-6}	1.56 × 10^{-5}
HPF10 (*z*=5)	Before	*Intensity* = (6.80 ± 0.57) × 10^8 *C* − (3.70 ± 1.74) × 10^3	0.9157	7.68 × 10^{-6}	1.54 × 10^{-5}
	After	*Intensity* = (4.33 ± 0.64) × 10^8 *C* + (3.81 ± 1.96) × 10^3	0.7771	1.36 × 10^{-5}	2.71 × 10^{-5}
HPF10 (*z*=4)	Before	*Intensity* = (8.00 ± 1.62) × 10^8 *C* + (2.59 ± 4.93) × 10^3	0.6521	1.85 × 10^{-5}	3.70 × 10^{-5}
	After	*Intensity* = (5.92 ± 1.12) × 10^8 *C* + (6.00 ± 3.40) × 10^3	0.6832	1.72 × 10^{-5}	3.45 × 10^{-5}

* Division by zero is undefined.

Table S2. Signal change factors of selected compounds in a solution of 5 mM sodium chloride. Each panel presents a different compound with an inset showing the linear range of the corresponding calibration curve.

**Compound**	**Signal change factor (I_{After} / I_{Before})**					
	**5 × 10^{-6} mol L^{-1}**	**1 × 10^{-5} mol L^{-1}**	**2 × 10^{-5} mol L^{-1}**	**4 × 10^{-5} mol L^{-1}**	**5 × 10^{-5} mol L^{-1}**	**1 × 10^{-4} mol L^{-1}**
Serine	*	*	*	*	*	*
Lysine	0.74 ± 0.08	1.03 ± 0.04	0.97 ± 0.02	0.95 ± 0.05	0.99 ± 0.00	1.05 ± 0.02
Glutamic acid	*	*	*	*	*	*
Acetaminophen	5.03 ± 0.25	12.89 ± 0.53	20.97 ± 1.87	38.82 ± 3.27	295.13 ± 17.04	40.14 ± 2.07
Histidine	0.97 ± 0.08	1.19 ± 0.02	1.22 ± 0.04	1.41 ± 0.06	1.73 ± 0.06	1.09 ± 0.02
Phenyalanine	3.96 ± 0.54	12.62 ± 1.03	18.86 ± 0.82	37.46 ± 1.43	35.33 ± 0.47	26.22 ± 0.05
Tryptophan	2.72 ± 0.23	9.15 ± 0.25	13.53 ± 0.86	21.49 ± 0.99	21.60 ± 0.34	16.31 ± 0.21
HPF1	0.92 ± 0.04	1.33 ± 0.09	5.51 ± 0.17	1.58 ± 0.09	2.56 ± 0.10	2.16 ± 0.07
HPF6 (*z*=6)	*	*	*	*	2.10 ± 0.19	2.42 ± 0.04
HPF6 (*z*=5)	*	4.58 ± 0.16	12.90 ± 0.86	6.77 ± 0.40	1.45 ± 0.08	1.47 ± 0.01
HPF6 (*z*=4)	*	1.47 ± 0.03	2.89 ± 0.17	1.88 ± 0.05	1.26 ± 0.01	1.23 ± 0.05
HPF6 (*z*=3)	*	0.65 ± 0.05	1.05 ± 0.05	1.17 ± 0.08	1.74 ± 0.06	1.66 ± 0.03
HPF10 (*z*=10)	*	*	*	*	*	*
HPF10 (*z*=9)	*	*	*	*	2.32 ± 0.00	2.63 ± 0.07
HPF10 (*z*=8)	*	*	*	17.78 ± 0.50	1.80 ± 0.01	1.96 ± 0.01
HPF10 (*z*=7)	*	9.14 ± 0.64	*	6.24 ± 0.18	1.31 ± 0.02	1.49 ± 0.02
HPF10 (*z*=6)	*	3.37 ± 0.12	13.91 ± 1.72	2.05 ± 0.07	0.91 ± 0.02	1.14 ± 0.03
HPF10 (*z*=5)	*	1.51 ± 0.12	3.77 ± 0.41	0.84 ± 0.05	0.76 ± 0.02	1.10 ± 0.06
HPF10 (*z*=4)	*	0.84 ± 0.08	1.50 ± 0.15	0.62 ± 0.10	1.03 ± 0.02	1.35 ± 0.05
Caffeine	2.06 ± 0.58	1.91 ± 0.45	1.90 ± 0.29	-	1.99 ± 0.22	2.47 ± 0.03
Caffeine[#]	1.39 ± 0.06	1.68 ± 0.15	2.38 ± 0.06	-	2.41 ± 0.04	2.55 ± 0.20

* Division by zero is undefined. The analyte signal was not observed before separation, and appeared after separation
Caffeine was prepared and analyzed separately, whilst all other compounds were prepared in the same solution
[#] Prepared in 1 mM sodium chloride solution

Table S3. Calibration plot parameters and limits of detection of caffeine (with and without isotope-labeled caffeine as an internal standard) in solutions of 1 mM and 5 mM sodium chloride, and concentration of caffeine in selected caffeinated beverages diluted 100 and 500 times. The LOD and LOQ values were calculated based on the slope and the intercept error provided by the Origin software.

**Solution**	**Internal standard use**	**Calibration equation (C / mol L^{-1})**	R^2	**LOD / mol L^{-1}**	**LOQ / mol L^{-1}**	**[Caffeine]$_{ED1-S}$ / g L^{-1}**		**[Caffeine]$_{ED1-SF}$ / g L^{-1}**		**[Caffeine]$_{ED2-S}$ / g L^{-1}**		**[Caffeine]$_{ED2-SF}$ / g L^{-1}**	
						**1:500 d.f.**	**1:100 d.f.**	**1:500 d.f.**	**1:100 d.f.**	**1:500 d.f.**	**1:100 d.f.**	**1:500 d.f.**	**1:100 d.f.**
1 mM NaCl	Non-normalized	*Intensity* = $(2.76 \pm 0.09) \times 10^9\, C + (3.82 \pm 1.00) \times 10^3$	0.9840	1.09×10^{-6}	3.64×10^{-6}	-	-	0.17 ± 0.02	0.24 ± 0.07	-	0.09 ± 0.09	-	0.09 ± 0.17
	Normalized	*Intensity* = $(1.04 \pm 0.01) \times 10^5\, C + (1.07 \pm 0.60) \times 10^{-2}$	0.9996	1.73×10^{-7}	5.77×10^{-7}	0.25 ± 0.01	0.10 ± 0.03	0.24 ± 0.00	0.27 ± 0.01	0.46 ± 0.06	0.49 ± 0.02	0.49 ± 0.01	0.41 ± 0.02
5 mM NaCl	Non-normalized	*Intensity* = $(1.85 \pm 0.13) \times 10^9\, C + (1.31 \pm 1.35) \times 10^3$	0.9386	2.19×10^{-6}	7.30×10^{-6}	-	-	-	0.22 ± 0.21	-	-	-	-
	Normalized	*Intensity* = $(1.09 \pm 0.01) \times 10^5\, C + (1.59 \pm 0.74) \times 10^{-2}$	0.9994	2.05×10^{-7}	6.83×10^{-7}	0.24 ± 0.01	0.09 ± 0.03	0.23 ± 0.00	0.26 ± 0.01	0.44 ± 0.06	0.47 ± 0.02	0.47 ± 0.01	0.39 ± 0.02
N/A	N/A	N/A	N/A	N/A	N/A	0.32**				0.54**		0.49**	

d.f. is dilution factor
** caffeine concentration labeled on the beverage packaging
Energy drink brand 1 (ED1) with sugar (ED1-S) and sugar free (ED1-SF)
Energy drink brand 2 (ED2) with sugar (ED2-S) and sugar free (ED2-SF)
